# Microbiological, biochemical and organoleptic properties of fermented-probiotic drink produced from camel milk

**Published:** 2017-12-15

**Authors:** Saideh Saljooghi, Ladan Mansouri-Najand, Hadi Ebrahimnejad, Farideh Doostan, Nasrin Askari

**Affiliations:** 1 *Graduated Student, Faculty of Veterinary Medicine, Shahid Bahonar University of Kerman, Kerman, Iran;*; 2 * Department of Food Hygiene and Public Health, * *Faculty of Veterinary Medicine, * *Shahid Bahonar University of Kerman, Kerman, Iran; *; 3 * Department of Nutrition, Faculty of Health, Kerman University of Medical Sciences, Kerman, Iran; *; 4 * Department of Pathobiology, Faculty of Veterinary Medicine, Shahid Bahonar University of Kerman, Kerman, Iran.*

**Keywords:** Biochemical properties, Biphidobacterum biphidum, Camel milk, Lactobacillus acidophilus, Sterptococcus thermophilus

## Abstract

The microbiological and biochemical changes occurred during the fermentation of camel milk inoculated by three selected bacterial starter, were investigated as well as the sensory evaluation of the product. Milk samples were collected from camel herds of southeastern of Iran. Chr. Hansen ABT-10 starter including *Lactobacillus acidophillus, Biphidobacterum biphidum *and* Sterptococcus thermophilus* in ratio of 0.50 g per 100 mL of camel milk was added. This fermented product was examined at the 0, 3^rd^, 6^th^ and 9^th^ days for microbiological, biochemical and sensory evaluations. The results showed the number of starter bacteria was maintained at least 10^6^ CFU mL^-1^ during nine test days. It was shown that it could be used as fermented-probiotic drink. The product did not show any microbial contamination. The acidity and protein amount of produced drink showed a significant (*p *< 0.05) increase in different test days. Fat, solids-not-fat and ash amount of the product showed significant differences at the ninths’ test day compared to the zero test day (*p* < 0.05). Organoleptic properties of product including flavor, color, odor, consistency, mouth feel and overall acceptance were significantly improved (*p *< 0.05). Therefore, the produced fermented–probiotic drink, in addition to keep maintenance and increased nutritional quantity value, was accepted by consumers in terms of organoleptic properties and it could be used as a healthy and functional drink.

## Introduction

Camels (*Camelus dromedaries*) belong to the family *Camelidae *and the sub-order *tylopoda*. There are nearly 150,000 dromedary camels living in the desert areas (South and Central) of Iran. This constitutes 0.56% of the world camel population and 3.80% of the Asian camel population.^1^ The majority of the camels are dromedary and scattered across the country in 14 provinces. The average daily milk yield of camels in Iran has found to be 1,880 kg annually.^2^

Camel milk is extremely popular and widely consumed in some area of Iran. In comparison to cow’s milk, camel milk has a high amount of humidity, protein, potassium, iron and vitamin C and lower amount of lactose.^3^ Different pharmaceutical properties are attributed to the camel milk and its products. The nutritional and therapeutic importance of fermented dairy products had been attributed to the use of lactic acid cultures in their manufacturing process and to numerous metabolites and enzymes produced that possess some therapeutic benefits.^4^

In Asian and African countries, different starters with different names are used. For example it is called Gariss in Sudan. It is prepared by fermenting the camel milk in large skin bags which contains a large quantity of a previously soured product.^5^ Another kind in East Africa, Kenya and Somalia is known as Suusac. It is prepared by fermenting fresh camel milk in a pre-smoked gourd naturally at ambient temperature (26 - 29 ˚C) for 1 to 2 days.^6^ Similarly in Kazakhstan is called Shubat and Chal in Turkey.

Other researchers reported that cheese made from 100% camel milk has lower yield and lower component recovery than cheese made from cow milk.^7^ They observed that camel milk failed to form gel like structure after 18 hr incubation with lactic acid culture and this was attributed to the presence of antibacterial factors such as lysozymes, lactoferrin immunoglobulin in camel milk. The consistency of fermented milk, under lab conditions, was thin because of the problem associated with milk coagulation. Thus, producing fermented camel milk products with high consistency due to this problem is difficult.^8^

Considering that Kerman is located on the edge of the desert and in terms of the number of camels stands on the second place of Iran, the fermented drinks from camel milk as a commercial product in this region should be noted. High milk camel breeds, can be replaced by other breeds in this region. Fermented camel milk in tropical area is considered as a desirable drink. The replacement of this useful drink in these regions or Iran needs more researches on this topic. Therefore, the present study was conducted to investigate the microbiological, biochemical, organoleptic changes in fermented camel milk by selected bacterial starter.

## Materials and Methods

**Preparation of fermented milk. **The milk samples were collected from camel herds in southeastern of Iran during May to October 2014. Samples were transported to the laboratory adjacent to ice. Milk was immediately cooled and kept at 5 ± 1 ˚C during transportation to the laboratory. The milk sample was heated at 95 ˚C for 15 minutes and then cooled to the inoculation temperature (43 ˚C).^8^ The Chr. Hansen ABT-10 starter (Chr. Hansen, Hørsholm, Denmark) in ratio of 0.50 g per 100 mL of camel milk was used. After that the samples were incubated at 43 ˚C for 5 hr to reach to the proper acidity (11 g L^-1^). Samples were cooled in the refrigerator for 12 to 18 hr to get better taste. This produced fermented sample was homogenized. Then all tests were done on the zero, third, sixth and ninth days of the study. Samples (50 mL) were taken in sterile bags aseptically for microbiological, biochemical and organoleptic tests. The experiment was repeated three times.

**Microbiological analysis. **Fermented camel milk samples (11 mL) were homogenized for one minute in 99 mL (1/10) of a sterile solution of 0.10% (w/v) peptone water (Oxoid, Cheshire, UK) using a Stomacher Lab blender (model 400; Seward Laboratory, London, UK). From these samples serial dilutions were prepared in sterile 0.10% peptone water. The microorganism counts were carried out by the pour plate method with duplicate plating on different selective agar media.^9^

The coliforms were estimated in duplicate pour plates of violet red bile agar medium (Oxoid) and the plates were overlaid after solidification with 3 to 4 mL of additional violet red bile agar. All plates were incubated in an inverted position at 35 ± 1 ˚C for 18 to 24 hr.^7^

The microbial count was performed for *Staphylococcus* by Baird Parker medium (Merck, Darmstadt, Germany) and surface culture method. The yeasts and molds were counted on acidified potato dextrose agar (Oxoid) which were acidified by addition of the proper amount of sterile 10% tartaric acid (Merck), then the plates were incubated at 22 ± 1 ˚C for three to seven days.^7^^,^^8^ The *Lactobacillus acidophillus* count was performed aerobically by De Man, Rogosa and Sharpe (MRS) agar medium (Merck) and bile hydroclorid (Merck).^8 ^The *Biphidobacterum biphidum *count was performed anaerobically by MRS agar medium hydroclorid cystein and mupyrocine (Merck).^8^

**Measurement of titratable acidity. **The titratable acidity (expressed as lactic acid %) was determined by titrating 10 mL of homogenized fermented camel milk with 0.10 N NaOH (Merck) to the phenolphthalein end point.

**Organoleptic evaluation. **Organoleptic test was performed by nine points’ Hedonic and questionnaire method.^10,11^ At first 20 mL of sample was poured into disposable containers and was presented to 20 students. Student’s opinions about flavor, color, odor, consistency, mouth feel and overall acceptance of the samples were collected. Individual’s selection was including very excellent, excellent, good, fairly good, medium, fairly bad, bad and very bad questionnaire marking. 

**Statistical analysis. **To analyze the chemical data, Bonferoni test and for organoleptic data Friedman test were used. A *p* value less than 0.05 was considered statistically significant.

## Results

The number of *L. acidophilus* bacteria colonies in the product did not show any significant decrease at the third, sixth and ninth days in comparison with the zero day (*p *< 0.05). The logarithm average of the colonies number at zero and ninth days were 3.11× 10^7^ and 1.65×10^7^, respectively, but, in all these samples the number of *L. acidophilus *was not less than 10^6^ CFU mL^-1^.

The number of *Bifidobacterium bacteria* colonies in the product showed a significant decrease at the 3^rd^, 6^th^ and 9^th^ days in comparison with the zero day (*p* < 0.05). 

Totally, according to statistics, the number of* Bifidobacterium* compared to *L. acidophilus* and the other lactic acid producing probiotic and its growth are lower in the product. Microbial count of *staphylococcus*, *Coliforms*, molds and yeast were negative during the different days. Probiotic bacteria maintenance results are shown in Table 1.

**Table 1:** Number of *Lactobacillus acidophilus*, *Biphidobacterium biphidum *colonies per mL of fermented-probiotic product at different test days. Data are presented as mean ± SE.

**Bacteria**	**0 day**	**3** ^rd^ ** day**	**6** ^th^ ** day**	**9** ^th^ ** day**
***Lactobacillus acidophilus***	3.11 × 10^7^ ± 6.70	2.66 × 10^7^ ± 6.70	2.70 × 10^7^ ± 6.90	1.65 × 10^7^ ± 6.80
***Biphidobacterium biphidum***	3.25 × 10^8^ ± 5.30	2.45 × 10^8^ ± 3.52	1.07 × 10^8^ ± 6.90	3.53 × 10^7^ ± 1.05

Chemical test results of camel milk and fermented-probiotic product are shown in Table 2. A significant difference between acidity, protein and fat amount of camel milk and product was observed on different test days (*p* < 0.05). Solids-not-fat amount of the product showed significant increase on the 9^th^ test day compared to the zero test day (*p* < 0.05) and did not show significant difference compared to the camel milk on the zero ,third, sixth and ninth test days.

**Table 2:** Chemical test results of camel milk and fermented-probiotic product at different test days.

**Chemical properties**	**Camel milk**	**0** **day**	**3** ^rd^ ** day**	**6** ^th ^ **day**	**9** ^th ^ **day**
**Acidity (g L** ^-1^ **)**	4.60	9.09	9.29	9.70	10.3
**Protein (%)**	2.55	3.98	4.15	4.50	4.70
**Fat (%)**	3.63	3.80	3.80	3.81	3.11
**Dry matter (%)**	8.77	9.06	9.42	9.54	9.94
**Solids-not-fat (%)**	5.14	5.26	5.62	5.72	6.15
**Ash (%)**	0.33	0.59	0.58	0.73	0.75

Ash amount of product showed significant increase on the zero, third, sixth and ninth days compared to the camel milk (*p* < 0.05) and did not show significant difference on the different days.

Organoleptic results are shown in Table 3. The product color score was not the same on different days. Odor, texture and thickness of product were the same on different days. Mouth feel and overall acceptance of product showed significant differences on different days (*p* < 0.05). The percentage of overall acceptance by consumers was 95% that was a good score. 

**Table 3:** Organoleptic properties of fermented-probiotic product of the camel milk at different test days. Data are presented as mean ± SE.

**Organoleptic properties **	**0 day**	**3** ^rd ^ **day**	**6** ^th^ ** day**	**9** ^th^ ** day**
**Flavor score**	3.04 ± 0.08^*^^a^	6.24 ±0.29^*^^b^	8.54 ± 0.02^*^^c^	8.18 ± 0.02^*^^d^
**Color score**	4.24 ± 0.20^†^^a^	6.40 ± 0.10^*^^b^	6.90 ± 0.10^†^^c^	6.80 ± 0.00^†^^c^
**Odor score**	6.28 ± 0.00^‡^^a^	6.58 ± 6.28^*^^b^	6.85 ± 6.20^†^^c^	6.58 ± 6.23 ^†^^b^
**Texture and thickness score**	6.80 ± 0.10 ^‡^^a^	6.58 ± 0.10^*^^a^	6.68 ± 0.00 ^†^^a^	6.63 ± 0.00^†^^a^
**Mouth feel score**	4.00 ± 0.01^†^^a^	6.85 ± 0.21^b^	7.68 ± 0.10^‡^^c^	7.48 ± 0.00^‡^^c^
**Total acceptance score**	7.48 ± 0.00^a^	6.72 ± 0.61^b^	7.39 ± 0.98 ^a^	7.66 ± 0.08^a^

*†‡ Different superscript symbols show significant difference (*p* < 0.05) between days 0, 3, 6 and 9, respectively.

abcd Different alphabetic letters show significant difference (*p* < 0.05) between different sensory characteristics.

## Discussion

Milk and dairy products play an important role in the food chain and their production are increasing.^12^ Probiotic term means “for life” in Greek language. Probiotic products contain beneficial bacteria that are resident in human gut and have beneficial effects on human health. In 2001, FAO and WHO reached at a common definition for probiotics. They are live micro-organisms with beneficial effects on the host health when used in enough amount.^13^

To develop production of probiotic products, it should be considered to choose strains based on functional criteria and basal environment. Sometimes selection of undesirable strain leads to inappropriate products.^14^

In the present study, fermented-probiotic product was produced by camel milk and three bacterial starters. Changes in maintenance probiotic bacteria, microbial contamination counts, chemical and organoleptic properties of the product were evaluated for nine days with three days interval. 

The number of *L. acidophilus* bacteria colonies in the product did not show any significant decrease at the 3^rd^, 6^th^ and 9^th^ days in comparison with the zero day (*p *< 0.05). The logarithm average of the colonies number at zero and ninth days were 3.11 × 10^7^ and 1.65 × 10^7^, respectively, however, in all these samples the number of *L. acidophilus *was not less than 10^6^ CFU mL^-1^.

Abdel Moneim *et al*. in a study on Garis showed that the dominant bacteria was lactic acid bacteria and the main genus (74.00%) was *Lactobacillu*s.^15^ Lore *et al*. showed that lactic acid bacteria logarithm was 6.80 per mL and the main genus was *Lactobacillus*.^6 ^Based on the previous finding decreased number of *Lactobacillus* is due to acid damage.^14^ Some studies showed that* Lactobacillus* in fermented ultra-high temperature processing (UHT) at the refrigerator temperature (4 ˚C) is remained stable and active and sometimes has a growing trend.^14,16^ The number of *Bifidobacterium bacteria* colonies in the product showed a significant decrease at the 3^rd^ , 6^th^ and 9^th^ days in comparison with the zero day (*p* < 0.05). Totally, according to statistics, the number of* Bifidobacterium* compared to* L. acidophilus* and the other acid producing probiotic and its growth are lower in the product.

In general, many factors affect probiotic bacteria life in fermented milk. The factors such as pH reducion, incubation temperature and oxygen presence has been known.^15 ^For example, *Bifidobacterium* is more sensitive to oxygen, high acidity and low pH.^17^

Based on the results of the present study, changes of the product acidity had an increasing trend on different days compared to camel milk (*p* < 0.05). The increasing acidity in camel milk and probiotic-fermented product on the zero, third, sixth and ninth days were 4.60, 9.10, 9.30, 9.71 and 10.31 g L^-1^, respectively. This could be due to acid production by *L. acidophillus* probiotic bacteria. 

In present study, the protein content of the product had an increasing amount on different test days and was significant more than camel milk (*p* < 0.05) and that was probably due to the protein production related to bacteria cells (single-cell protein).

The fat content of product did not show significant difference on different days and in comparison with the camel milk. The solid content of product showed a significant difference on ninth day in compared to the zero day and camel milk (*p* < 0.05). The solid content of product on the ninth, zero days and milk was 9.94, 9.07 and 8.77%, respectively; that was probably due to increased protein and partial reduction of water during the product storage. However, solid content of camel milk did not show significant difference compared to the product on the zero, third, sixth days and product solid content did not show significant difference on different days with each other. 

Ash content of product on the zero, third, sixth days was more than camel milk and showed a significant increase (*p* < 0.05), however, did not show a significant increase in different test days.

Chemical compound of camel milk and Chall was compared by Grigoryants in 2012. The results showed that Chall and milk fat content was equal and was 4.30% that showed the lower amount of lactose, ash and ascorbic acid in milk rather than Chall.^16^

Evaluation of organoleptic changes in the product showed that its flavor (*p* < 0.05), color, feeling and overall acceptance were better by the time passing (*p* < 0.05) but smell score, texture and thickness did not show a significant difference on different test days.

Abdel Moneim *et al. *in a study on fermented milk using yogurt bacteria showed that the smell, thickness and overall acceptance of product had significant score in comparison with other starters.^15^ The thickness of all fermented camel milk products had aqueous, fragile and heterogeneous state^16^^,^^18^ that was similar to the present study results.

In conclusion, this fermented–probiotic drink, in addition to keeping maintenance and increasing nutritional quantity value, was accepted by consumers in terms of organoleptic properties and it could be used as a healthy and functional drink. Kerman is located next to the desert area and in terms of the number of camels, is the second place of Iran. Therefore, the fermented drinks from camel milk as a desirable product in this region should be considered commercially. 
